# Prismatic Image Relocation in Macular Disease: Effects on Visual Function and the Preferred Retinal Locus—A Systematic Review and Meta-Analysis

**DOI:** 10.3390/jcm15218322

**Published:** 2026-10-26

**Authors:** Francisco Javier Povedano-Montero, Rut González-Jiménez, Ricardo Bernárdez-Vilaboa, Sofia Bernardez-Sánchez, Juan E. Cedrún-Sánchez

**Affiliations:** 1 Optometry and Vision Department, Faculty of Optics and Optometry, Complutense University of Madrid, 28037 Madrid, Spainricardob@ucm.es (R.B.-V.); jcedrun@ucm.es (J.E.C.-S.); 2 Instituto de Investigación Sanitaria Hospital 12 de Octubre (imas12), 28041 Madrid, Spain; 3 Department of Chemistry and Biochemistry, Universidad CEU San Pablo, 28668 Madrid, Spain; 4 Applied Vision Research Group, Faculty of Optics and Optometry, Universidad Complutense de Madrid, 28037 Madrid, Spain

**Keywords:** macular degeneration, prism spectacles, image relocation, preferred retinal locus, low vision rehabilitation, visual acuity, meta-analysis

## Abstract

**Purpose:** To evaluate the effectiveness of prismatic image relocation for visual rehabilitation in patients with macular disease and central vision loss, focusing on visual acuity, reading-related outcomes, preferred retinal locus (PRL) behaviour, fixation, and functional outcomes. **Methods:** A systematic review and meta-analysis was conducted according to PRISMA 2020 and prospectively registered in PROSPERO (CRD420261500738). Scopus, Web of Science Core Collection, PubMed/MEDLINE, and CENTRAL were searched from inception, with searches conducted between 2 and 15 September 2026. Randomized, non-randomized comparative, pre-post, and case-series studies evaluating prism-based image relocation were eligible. Risk of bias was assessed using RoB 2, ROBINS-I, or the appropriate Joanna Briggs Institute tools. Random-effects meta-analysis was performed when studies were sufficiently comparable. **Results:** Eleven studies met the inclusion criteria. Two randomized controlled trials provided comparable data for an exploratory meta-analysis of distance visual acuity. The pooled point estimate favoured prism correction (MD, −0.218 logMAR), but the Hartung–Knapp-adjusted 95% confidence interval was extremely wide (−2.188 to 1.753) and included the null effect, with substantial heterogeneity (I^2^ = 94.5%). The certainty of evidence for the primary outcome was rated as very low. Sensitivity analyses did not materially strengthen the evidence. Observational studies frequently reported favourable visual or functional outcomes, but several were affected by important risks of selection bias, confounding, or absence of appropriate control groups. Evidence concerning PRL relocation and fixation behaviour was sparse, heterogeneous, predominantly non-randomized, and unsuitable for quantitative synthesis. **Conclusions:** Current evidence is insufficient to establish the routine efficacy of prismatic image relocation for improving distance visual acuity in patients with macular disease and central vision loss. Prism correction may nevertheless provide visual or functional benefit in selected individuals and may be considered as an individualized component of structured low-vision rehabilitation.

## 1. Introduction

Macular diseases are a major cause of central vision loss and can substantially impair visual acuity, reading performance, face recognition, and other visually demanding activities. When foveal function becomes severely compromised, many patients develop an eccentric retinal area for fixation, commonly referred to as the preferred retinal locus (PRL). The PRL acts as an alternative fixation locus and may partially compensate for the loss of foveal function, although its location, stability, and functional efficiency vary considerably among individuals [1,2,3].

The naturally adopted PRL does not necessarily correspond to the retinal area with the highest residual visual acuity or sensitivity. Instead, its development may be influenced by factors such as proximity to the former fovea, lesion configuration, fixation behaviour, binocular viewing conditions, and disease progression [4,5,6,7]. Consequently, the spontaneous PRL may not always provide the most efficient use of the remaining retinal function. This observation has led to the development of rehabilitation strategies aimed at improving eccentric viewing or directing visual information toward more functional retinal areas [8,9,10].

Prismatic image relocation represents one such approach. By optically displacing the image away from the central scotoma and toward functioning eccentric retina, prisms may facilitate the use of an existing PRL or, potentially, promote fixation at a more advantageous retinal locus. Different approaches have been described, including yoked prisms, unilateral or bilateral prismatic corrections, and Fresnel prisms, with considerable variation in prism power, base orientation, and methods used to identify the target retinal area [11,12,13,14,15]. The concept is also closely related to potential visual acuity, since prismatic correction may theoretically allow patients to approach the level of visual performance available at the most functional residual retinal locus [11,16].

Despite this physiological and optical rationale, the clinical evidence remains inconsistent. Some observational studies and controlled trials have reported improvements in distance or near visual acuity, reading performance, fixation behaviour, or vision-related quality of life following prismatic correction [11,12,13,14]. In contrast, other investigations, including controlled studies, have failed to demonstrate a clear advantage over conventional spectacles or placebo correction [17,18]. Moreover, changes in visual function do not necessarily imply a stable relocation of the PRL, and microperimetric studies have suggested that prism-induced changes in fixation location may be small, variable, or unpredictable [18,19].

Previous reviews have addressed low-vision rehabilitation, eccentric viewing, or the use of prisms in macular disease, but the available evidence remains heterogeneous with respect to study design, prism prescription, underlying macular disorder, outcome definition, and follow-up [19,20,21]. A comprehensive synthesis specifically focused on prismatic image relocation is therefore needed to determine whether this intervention produces measurable visual or functional benefits and to clarify its relationship with PRL behaviour.

The aim of this systematic review and meta-analysis was to evaluate the effectiveness of prismatic image relocation for visual rehabilitation in patients with macular disease and central vision loss, with particular emphasis on visual acuity, reading-related outcomes, fixation characteristics, preferred retinal locus behaviour, and patient-reported functional outcomes.

## 2. Materials and Methods

### 2.1. Study Design and Protocol

A systematic review and meta-analysis was conducted to evaluate the effectiveness of prismatic image relocation as a visual rehabilitation strategy in patients with macular disease and central vision loss. The review was designed and conducted in accordance with the PRISMA 2020 [22] recommendations.

The review protocol was prospectively registered in PROSPERO (CRD420261500738; registered on 9 September 2026) before study screening and defined the population of interest, eligible interventions, outcomes, and planned methods of synthesis. The target population comprised patients with macular disease associated with central scotoma, eccentric fixation, or use of a preferred retinal locus (PRL), with no age restrictions.

Reporting of this systematic review and meta-analysis was guided by the PRISMA 2020 statement. The completed PRISMA 2020 checklist is provided in Supplementary Table S1. Any deviations from the registered protocol and post hoc analytical decisions arising during the review process were documented and justified and are described in Section 2.10.

### 2.2. Information Sources and Search Strategy

A systematic literature search was conducted across the prespecified databases between 2 and 15 September 2026, covering each database from inception to the date of search, with no restrictions on publication date or language. The electronic databases searched were Scopus, Web of Science Core Collection, PubMed/MEDLINE, and the Cochrane Central Register of Controlled Trials (CENTRAL). These information sources were prespecified in the registered PROSPERO protocol and were selected to provide complementary coverage of biomedical, controlled-trial, and multidisciplinary literature.

The search strategy combined terms related to prismatic intervention with terms describing macular disease, central vision loss, eccentric viewing, and the preferred retinal locus. The core search structure was:

(“prism*” OR “prismatic correction” OR “prism spectacle*” OR “yoked prism*” OR “Fresnel prism*” OR “image relocation” OR “relocating prism*”)

AND

(“macular degeneration” OR “age-related macular degeneration” OR AMD OR “macular disease*” OR maculopath* OR “macular dystroph*” OR Stargardt OR “central scotoma*” OR “central vision loss” OR “preferred retinal locus” OR PRL OR “eccentric fixation” OR “eccentric viewing”).

The same conceptual search structure was used across databases and was adapted to the syntax and searchable fields supported by each platform. In Scopus, the terms were searched using TITLE-ABS-KEY fields, whereas Topic (TS) fields were used in Web of Science Core Collection. Equivalent Boolean combinations of the same search concepts were used in PubMed/MEDLINE and CENTRAL according to the search fields available in each database. The database-specific adaptations of the search strategy and the number of records retrieved from each source are provided in Supplementary Table S2.

The searches retrieved 705 records from Scopus, 224 from Web of Science Core Collection, 190 from PubMed/MEDLINE, and 48 from CENTRAL. Records were merged into a single master database and deduplicated hierarchically using DOI, PMID, database-specific identifiers, and, when these were unavailable, combinations of title, authors, publication year, and source.

Reference lists of eligible studies and relevant reviews were examined, and forward citation searching of the included studies was performed using Web of Science and/or Scopus to identify additional potentially eligible reports.

Embase and CINAHL were not included in the prospectively registered search strategy. The selected database set combined biomedical, controlled-trial, and multidisciplinary citation-indexed sources, together with backward and forward citation searching. Because study identification and screening had been completed according to the prespecified protocol, the database set was not expanded post hoc during peer review. The possibility that relevant reports indexed exclusively in databases not searched may have been missed is acknowledged as a limitation.

### 2.3. Eligibility Criteria

Studies were considered eligible if they included patients with age-related macular degeneration, myopic maculopathy, macular dystrophies, Stargardt disease, or other macular disorders associated with central vision loss, central scotoma, eccentric fixation, or use of a PRL. Studies involving mixed populations were eligible only when data for patients with macular disease could be identified separately.

Eligible interventions included optical image relocation using prisms, such as yoked prisms, prismatic spectacles, unilateral or bilateral prismatic corrections, and Fresnel prisms, provided that the purpose of the intervention was to redirect the retinal image toward a functional retinal area, PRL, or eccentric fixation locus. Combined low-vision interventions were considered only when the prismatic component was clearly defined or its contribution could be evaluated separately.

Randomized controlled trials, non-randomized controlled studies, prospective or retrospective cohort studies, pre-post studies without a control group, and case series providing relevant quantitative data were eligible. Single case reports, narrative reviews, systematic reviews, meta-analyses, editorials, letters, theoretical optical studies, and reports without original clinical data were excluded.

Studies in which prisms were used exclusively for strabismus, diplopia, hemianopia, neurological visual field defects, diagnostic purposes, or peripheral field expansion without a macular rehabilitation objective were also excluded.

Conference abstracts and other incomplete reports were eligible only when they contained sufficient information to establish study eligibility and extract at least one prespecified outcome; otherwise, they were not included in quantitative synthesis.

No mandatory comparator was required. Eligible comparators included placebo or sham spectacles, spectacles without prism, conventional low-vision rehabilitation, alternative rehabilitation strategies, or no intervention.

### 2.4. Study Selection

All retrieved records were merged into a single database before screening. After removal of duplicates, 782 unique records remained.

Title and abstract screening was performed independently by two reviewers. Each record was classified as Include, Exclude, or Uncertain. Disagreements and uncertain records were subsequently reassessed, and unresolved cases were adjudicated by a third reviewer.

Reports judged potentially eligible after title and abstract screening were assessed at full-text level whenever the complete report could be retrieved. Reasons for exclusion at this stage were documented individually. When a full-text report could not be obtained, a sufficiently detailed indexed abstract or another available report of the same study was considered only when it provided enough information to establish eligibility. Such studies were explicitly flagged as being based on incomplete reporting, and data extraction was restricted to information directly reported in the available source; unpublished values were not imputed. Reports providing insufficient information to establish eligibility or extract prespecified outcome data were not included in quantitative synthesis. No study contributing to the primary meta-analysis relied exclusively on abstract-level data.

Trial registry entries and multiple reports corresponding to the same underlying study were linked and were not treated as independent studies.

**Figure 1:**
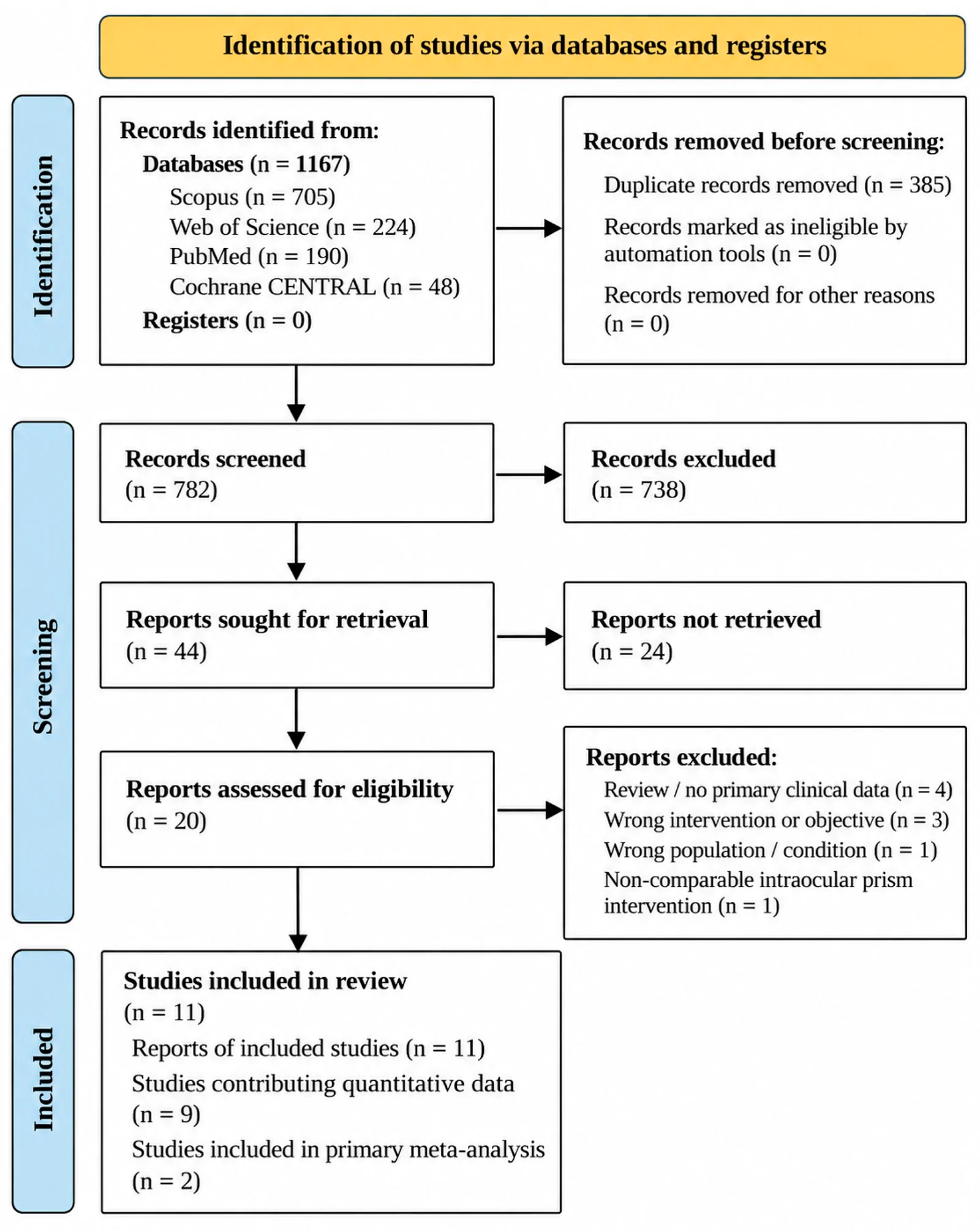
PRISMA 2020 flow diagram of the study selection process. When full reports could not be retrieved, sufficiently detailed indexed abstracts or other available reports were considered for eligibility assessment when they contained adequate information, as prespecified in the review methods.

The overall study identification and selection process is summarized in Figure 1.

Records were deduplicated across databases using DOI, PMID, title, authors, and publication year. When full-text reports could not be retrieved, sufficiently detailed abstracts were used only when they provided enough information to establish eligibility and, where applicable, extract prespecified outcome data. One trial registry entry corresponded to a published article and was not treated as an independent study.

### 2.5. Data Extraction

Data were extracted using a standardized form developed specifically for this review. Data extraction was performed independently by two reviewers. Disagreements were resolved by consensus and, when necessary, by consultation with a third reviewer.

For each study, the following information was recorded: authors, publication year, report status (full report or abstract/incomplete report), study design, macular disease phenotype and severity, sample size and number of participants per group, baseline distance visual acuity, scotoma characteristics and baseline PRL characteristics when reported, comparator, and duration of follow-up.

Detailed intervention characteristics were also extracted, including prism type, power and base direction, monocular or binocular application, fixed versus individualized prescription, intended retinal target or angular displacement when reported, method used to identify the target retinal locus, and whether the expected displacement was subsequently confirmed using fundus-tracked testing or microperimetry. Concomitant refractive correction, magnification, low-vision rehabilitation, or training components were also recorded. Binocular status, including ocular dominance, suppression, and stereopsis, was extracted when available.

For each outcome, the definition, measurement instrument or testing procedure, assessment time point, sample size, event counts, means, standard deviations, baseline and post-intervention values, change-from-baseline values, and available measures of precision were extracted whenever reported. Outcome definitions and measurement methods were also recorded to determine whether quantitative pooling was clinically and methodologically appropriate.

Data were preferentially extracted from the full-text article. When only a sufficiently detailed abstract was available, extraction was restricted to information explicitly reported in the abstract, without imputing unpublished values.

Potential overlap between study populations was also assessed by comparing authorship, study setting, recruitment period, sample characteristics, and intervention details. Studies suspected of including overlapping participants were not considered statistically independent unless cohort independence could be established.

### 2.6. Risk of Bias Assessment

Risk of bias was independently assessed by two reviewers using design-specific validated tools. Disagreements were resolved by consensus or, when necessary, by consultation with a third reviewer.

Randomized controlled trials with genuinely random allocation were evaluated using the Cochrane Risk of Bias 2 (RoB 2) tool, considering bias arising from the randomization process, deviations from intended interventions, missing outcome data, outcome measurement, and selection of the reported result. Studies using predictable or quasi-random allocation procedures, including allocation based on odd/even case number, were not treated as genuinely randomized trials and were assessed using ROBINS-I. Other non-randomized comparative intervention studies were also assessed using ROBINS-I, covering bias due to confounding, participant selection, intervention classification, deviations from intended interventions, missing data, outcome measurement, and selection of the reported result.

For uncontrolled observational, pre-post, and case-series designs, the corresponding Joanna Briggs Institute (JBI) critical appraisal checklists were used. JBI assessments are reported at the individual-item level as yes, no, unclear, or not applicable, as appropriate. No numerical score or synthetic overall JBI risk-of-bias category was assigned.

Risk-of-bias judgments were based on the information available in the full report whenever possible. When only an abstract or incomplete report was available, the assessment was considered provisional because relevant methodological information, including allocation procedures, masking, attrition, and selective reporting, could not always be verified.

### 2.7. Reporting Bias and Certainty of Evidence

Risk of bias due to missing results was assessed by considering selective outcome reporting and the availability of prespecified outcomes when study protocols or trial registrations were available. Assessment of small-study effects and publication bias using funnel plots and statistical tests for asymmetry was planned only when at least 10 studies contributed to the same meta-analysis.

The certainty of evidence for the primary outcome was assessed using the GRADE approach, considering risk of bias, inconsistency, indirectness, imprecision, and publication bias. Randomized evidence initially received a high-certainty rating. Any downgrading was recorded explicitly by domain, including the number of levels and the rationale for each decision. Certainty was classified as high, moderate, low, or very low. Domain-level judgments and their justification are provided in Supplementary Table S3 and are also summarized in Section 3.4.

### 2.8. Outcomes

The primary outcome was distance visual acuity, preferably expressed in logMAR units. For comparative studies, between-group effects at clinically comparable follow-up times were prioritized. Baseline-adjusted treatment-effect estimates, such as ANCOVA estimates, were preferred when reported and when they were directly comparable across studies. When adjusted estimates were unavailable or not comparable across trials, change-from-baseline contrasts were used when methodologically appropriate; otherwise, final-value between-group differences were used, with baseline visual acuity explicitly examined when interpreting the result. Alternative available effect estimates were evaluated in sensitivity analyses whenever they could be derived without unsupported assumptions.

Uncontrolled pre-post studies were analyzed separately. When visual acuity was reported using another scale, conversion to logMAR was undertaken only when methodologically appropriate. No threshold for a clinically meaningful change in distance visual acuity was prespecified in the registered protocol; therefore, no post hoc efficacy threshold was introduced.

Secondary outcomes included near visual acuity, reading performance, reading speed, critical print size, contrast sensitivity, fixation stability, PRL location and displacement, microperimetric parameters, vision-related quality of life, treatment tolerance, adherence, and the proportion of participants showing functional or visual improvement.

To make the feasibility of quantitative synthesis transparent, secondary outcomes were mapped by study according to outcome definition, measurement instrument or testing procedure, and assessment time point. This study-by-outcome matrix is provided in Supplementary Table S4.

### 2.9. Statistical Analysis

Meta-analysis was undertaken when at least two studies were considered sufficiently comparable in terms of population, intervention, comparator, study design, outcome definition, and follow-up. Because clinical and methodological heterogeneity was anticipated, random-effects models were used for quantitative synthesis.

For continuous outcomes measured on the same scale, mean differences (MDs) with 95% confidence intervals (CIs) were calculated. Standardized mean differences (SMDs) were considered when conceptually comparable outcomes were measured using different scales. Dichotomous outcomes were summarized using risk ratios (RRs) with 95% CIs. Controlled studies and uncontrolled pre-post studies were analyzed separately, and clinically distinct outcomes were not combined within a single pooled estimate.

The primary quantitative synthesis was restricted to genuinely randomized controlled trials reporting clinically comparable distance visual-acuity outcomes. Quasi-randomized and non-randomized comparative studies were synthesized separately or examined in sensitivity analyses. Studies with unresolved reporting inconsistencies or serious/critical risk-of-bias concerns were not included in the primary pooled estimate.

For the primary random-effects analysis, between-study variance (τ^2^) was estimated using restricted maximum likelihood (REML). Confidence intervals around the pooled effect were calculated using a Hartung–Knapp adjustment to better reflect the uncertainty associated with the very small number of contributing studies. Conventional REML estimates using normal-approximation confidence intervals and the original DerSimonian–Laird model were examined as sensitivity analyses to assess the influence of the statistical method on the conclusions.

Statistical heterogeneity was characterized using τ^2^, I^2^, and Cochran’s Q. Given that only two studies contributed to the primary meta-analysis, these measures were interpreted descriptively and were not regarded as precise estimates of between-study heterogeneity. A prediction interval was not calculated because the number of studies was insufficient for a meaningful estimate.

Sensitivity analyses examined the robustness of the findings to the analytical method and to alternative study or effect-estimate inclusion decisions. These included, where derivable, alternative adjusted or change-from-baseline estimates for Smith et al. [17]; inclusion of the numerical values reported by Tanveer et al. [23] despite the internal reporting inconsistency; and inclusion of the non-randomized comparative study by Al-Karmi and Markowitz [12]. When quantitative pooling was not clinically or methodologically appropriate, findings were summarized using structured narrative synthesis.

All statistical analyses were performed using Python (version 3.13.5; Python Software Foundation, Wilmington, DE, USA). Statistical tests were two-sided, with a nominal significance level of 0.05. Given the very small number of studies contributing to the primary meta-analysis, the pooled estimate was interpreted as exploratory and was not used as the sole basis for conclusions regarding treatment efficacy.

### 2.10. Protocol Deviations and Post Hoc Analytical Decisions

Two post-registration methodological decisions require explicit reporting. First, Rosenberg et al. [24] was reclassified as a quasi-randomized rather than genuinely randomized study because allocation was based on odd/even case number, a predictable assignment procedure. The study therefore remained eligible for the systematic review but was assessed using ROBINS-I and was not considered part of the genuinely randomized evidence base.

Second, exclusion of Tanveer et al. [23] from the primary meta-analysis because of an internal inconsistency between the published numerical results and the authors’ narrative interpretation was not prespecified in the PROSPERO protocol and was treated as a post hoc analytical decision. The published numerical values were retained for sensitivity analysis rather than being discarded. An attempt was made to obtain clarification from the study authors regarding this discrepancy, but clarification could not be obtained. Accordingly, the published numerical values were retained only for sensitivity analysis, and the study was not included in the primary meta-analysis because the correct effect estimate could not be established with sufficient confidence from the published report.

Although the registered protocol stated that study authors would not be contacted for additional information, following a request raised during peer review, a single attempt was made to contact the authors of Tanveer et al. [23] to clarify the internal inconsistency between the published numerical results and their narrative interpretation. This post-review procedural deviation was limited to clarification of the published data and did not affect the eligibility criteria, outcome definitions, or the prespecified synthesis framework.

No changes were made to the eligibility criteria, primary outcome, prespecified database set, or the requirement that quantitative synthesis should be undertaken only when at least two clinically and methodologically comparable studies reported the same outcome.

## 3. Results

### 3.1. Study Selection Results

The database searches identified 1167 records, including 705 from Scopus, 224 from Web of Science Core Collection, 190 from PubMed/MEDLINE, and 48 from the Cochrane Library/CENTRAL. After merging the retrieved records and removing 385 duplicates, 782 unique records remained for title and abstract screening. Of these, 738 were excluded, and 44 reports were sought for retrieval. Full reports could not be obtained for 24 records, whereas 20 reports were retrieved and assessed for eligibility. When the complete report was unavailable, sufficiently informative indexed abstracts or other available reports were additionally considered according to the prespecified eligibility criteria. Five of the studies ultimately included in the qualitative synthesis were assessed from abstract-level or otherwise incomplete reports; none contributed to the primary meta-analysis.

After completion of the eligibility assessment, 11 studies met the inclusion criteria and were retained for the systematic review. Nine retrieved reports were excluded: four were reviews or reports without primary clinical data [10,19,25,26], three evaluated interventions or objectives outside the scope of prism-based macular rehabilitation [27,28,29], one involved an ineligible clinical population or rehabilitation context [30], and one evaluated a non-comparable intraocular prism intervention [31]. The complete study-selection process is presented in Figure 1.

### 3.2. Study Characteristics

Eleven studies met the eligibility criteria and were included in the systematic review. The 11 included studies were Rosenberg et al. [24], Verezen et al. [32], Bertrand et al. [33], Parodi et al. [14], Vettard et al. [15], Smith et al. [17], Al-Karmi and Markowitz [12], Reyes et al. [13], Lewerenz et al. [18], Markowitz et al. [11], and Tanveer et al. [23].

The evidence base comprised three genuinely randomized controlled trials [14,17,23], one quasi-randomized comparative study [24], one non-randomized comparative study [12], and observational, pre–post, case-series, or within-subject studies [11,13,15,18,32,33], reflecting substantial methodological heterogeneity. Most studies investigated patients with age-related macular degeneration and central vision loss, whereas a smaller number included broader populations with macular dysfunction or central scotoma.

Prism interventions differed substantially in power, base orientation, monocular versus binocular application, degree of individualization, method used to select the retinal target, and accompanying low-vision care. Follow-up ranged from immediate assessment after prism placement to long-term observational follow-up. Five included studies were assessed using abstract-level or otherwise incomplete reports [13,15,18,32,33], whereas the studies contributing to the primary meta-analysis were available as complete reports. Detailed study characteristics and report status are provided in Supplementary Table S5. The availability, definition, timing, and suitability for quantitative synthesis of the outcomes reported by each study are summarized in Supplementary Table S4.

### 3.3. Risk of Bias and Methodological Quality

Risk of bias varied substantially across the included studies and was closely related to study design (Table 1). Among the genuinely randomized controlled trials assessed using RoB 2, Parodi et al. [14] and Smith et al. [17] were judged as having some concerns, whereas Tanveer et al. [23] was judged to be at high risk of bias. The principal concerns in Parodi et al. [14] related to limited reporting of allocation and masking procedures and post-randomization discontinuations, whereas in Smith et al. [17] the main concern related to missing outcome data. Tanveer et al. [23] presented additional concerns related to the randomization process, selective reporting, and the internal inconsistency between the numerical distance-visual-acuity data and the authors’ narrative interpretation.

Rosenberg et al. [24] was reclassified as a quasi-randomized study because allocation was based on odd/even case number and was therefore predictable. When assessed using ROBINS-I, the study was judged to be at serious risk of bias, principally because of the non-concealed allocation procedure and limitations in the control of potential confounding and selection processes. Al-Karmi and Markowitz [12], also assessed using ROBINS-I, was judged to be at critical risk of bias, primarily because prism prescription depended on the participant’s initial response to prism testing, making the intervention and comparison groups inherently different.

The remaining observational, pre–post, case-series, and within-subject studies were appraised using the appropriate JBI critical appraisal checklists. In accordance with JBI methodology, no synthetic overall risk-of-bias category was assigned. Item-level limitations included unclear consecutive or complete inclusion in several case series, absence of an independent concurrent control group in several studies, and incomplete reporting of follow-up or concomitant care. Detailed assessments are provided in Supplementary Tables S6–S8.

### 3.4. Effect of Prism Correction on Distance Visual Acuity

Two randomized controlled trials, Parodi et al. [14] and Smith et al. [17], provided sufficiently comparable data for an exploratory quantitative synthesis of distance visual acuity at approximately 3 months. Both compared prism correction with a non-prismatic control condition, reported distance visual acuity using the logMAR scale, and provided outcome data at a comparable follow-up interval. However, substantial differences existed in population characteristics, prism design, monocular versus binocular application, individualization of prism orientation, comparator design, and concomitant low-vision care. These characteristics are compared directly in Table 2.

As shown in Table 2, the two trials were comparable with respect to the primary outcome scale and follow-up interval but differed substantially in intervention design and clinical context. These differences were considered when interpreting the pooled estimate and the observed between-study heterogeneity.

**Figure 2:**
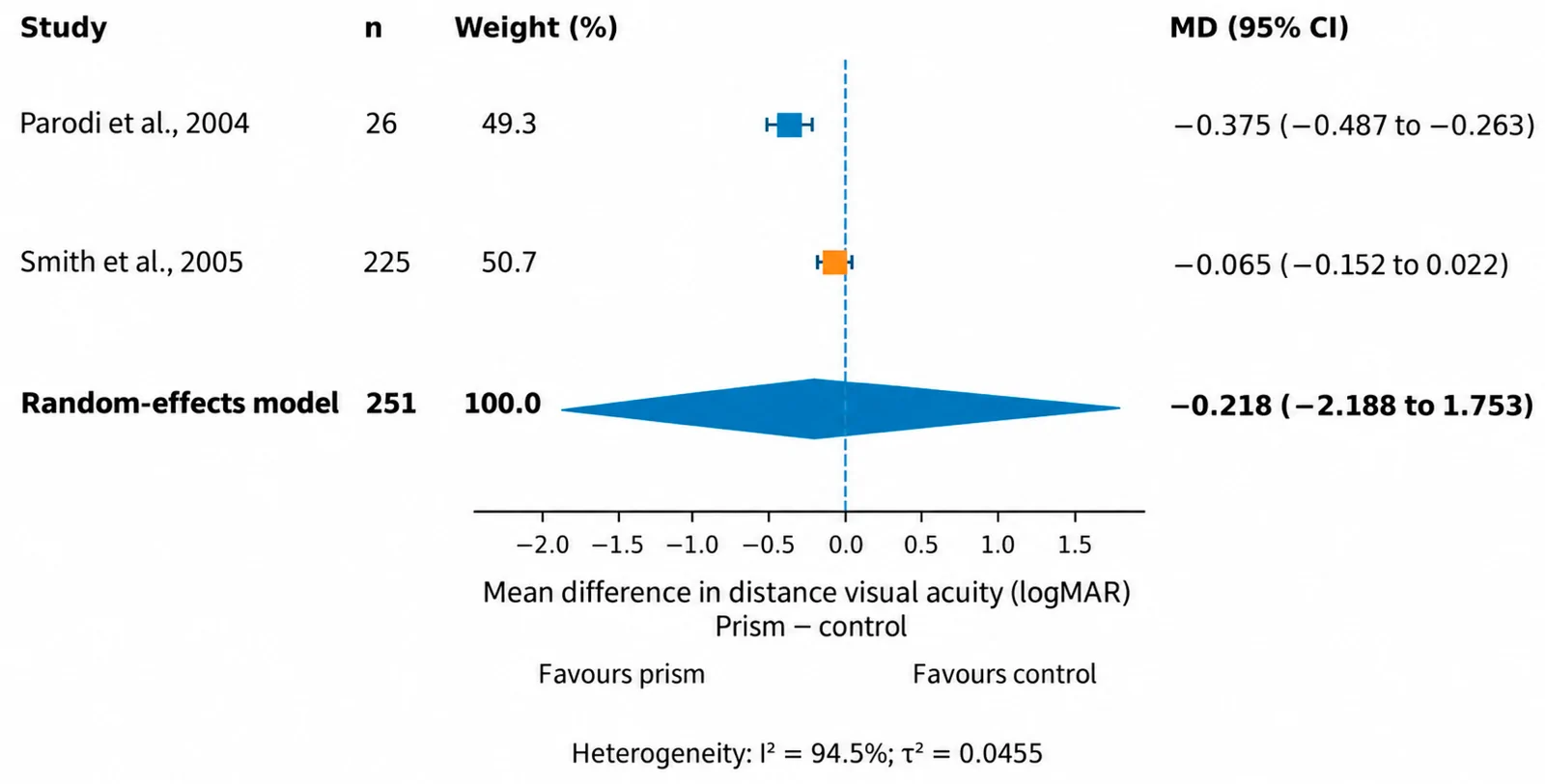
Forest plot of the exploratory primary meta-analysis evaluating prism spectacles versus non-prismatic control for distance visual acuity (logMAR) at approximately 3 months. The blue square represents Parodi et al. [14], and the orange square represents Smith et al. [17]. Square sizes are proportional to study weights, and horizontal lines indicate 95% confidence intervals (CIs). The blue diamond represents the pooled effect estimate, with its width indicating the Hartung–Knapp-adjusted 95% CI. The pooled estimate was calculated using a restricted maximum likelihood (REML) random-effects model. Negative values favour prism correction. Heterogeneity statistics (I^2^ and τ^2^) are shown for descriptive purposes. Because only two trials contributed to the analysis, the pooled estimate should be interpreted as exploratory.

The individual trial estimates differed markedly. Parodi et al. [14] showed a mean difference of −0.375 logMAR (95% CI, −0.487 to −0.263), whereas the combined active prism arms of Smith et al. [17] showed a mean difference of −0.065 logMAR (95% CI, −0.152 to 0.022) compared with placebo spectacles. The exploratory REML random-effects model yielded a pooled mean difference of −0.218 logMAR. However, the Hartung–Knapp-adjusted 95% confidence interval was extremely wide (−2.188 to 1.753), clearly including the null effect and reflecting the substantial uncertainty associated with a random-effects synthesis based on only two trials (Figure 2).

Statistical heterogeneity was substantial (I^2^ = 94.5%; τ^2^ = 0.0455), although both estimates are themselves unstable when derived from only two studies. The pooled point estimate corresponds approximately to 2.2 ETDRS lines in the direction favouring prism correction, but the extreme imprecision of the Hartung–Knapp interval precludes a meaningful conclusion regarding the magnitude or clinical importance of the effect. Accordingly, the pooled estimate should be regarded as exploratory and should not be interpreted independently of the individual trial results.

In Smith et al. [17], baseline distance visual acuity was 0.88 ± 0.33 logMAR in the custom-prism group, 0.89 ± 0.35 in the standard-prism group, and 0.94 ± 0.31 in the placebo group. The published baseline-adjusted ANCOVA estimates were −0.02 logMAR for both the custom-prism versus placebo comparison (95% CI, −0.07 to 0.02) and the standard-prism versus placebo comparison (95% CI, −0.06 to 0.03), with no overall treatment effect (*p* = 0.63). Because the two adjusted comparisons shared the same placebo group and the covariance required to derive a valid combined adjusted estimate was not reported, these ANCOVA estimates were not pooled with Parodi et al. [14]. without additional assumptions. Their near-null magnitude was nevertheless considered in the sensitivity interpretation.

The certainty of evidence for the effect of prism correction on distance visual acuity was rated as very low according to GRADE. Starting from high-certainty randomized evidence, certainty was downgraded by one level for risk of bias, one level for inconsistency, and one level for imprecision. No downgrade was applied for indirectness, and publication bias could not be formally assessed with only two contributing trials. The full domain-level assessment is provided in Supplementary Table S3.

### 3.5. Sensitivity Analyses

Sensitivity analyses confirmed that the conclusions were highly dependent on the uncertainty model but did not provide stronger evidence of a treatment effect. Using REML with the conventional normal approximation produced the same pooled point estimate as the primary analysis (MD, −0.218 logMAR; 95% CI, −0.522 to 0.086). The original DerSimonian–Laird analysis produced an essentially identical point estimate and between-study variance. Thus, changing the estimator had little effect on the pooled point estimate, whereas use of the Hartung–Knapp adjustment markedly increased the uncertainty around that estimate.

Tanveer et al. [23] was examined only in sensitivity analysis because the numerical distance-visual-acuity data were internally inconsistent with the authors’ narrative interpretation. At one month, the published table reported mean logMAR visual acuity of 0.65 ± 0.10 in the prism group and 0.47 ± 0.11 in the control group. Because lower logMAR values indicate better visual acuity, these numerical values imply a between-group effect favouring the control group (MD, +0.180 logMAR), whereas the article states that improvement in distance vision was significantly greater in the intervention group. No clarification could be obtained from the study authors.

**Figure 3:**
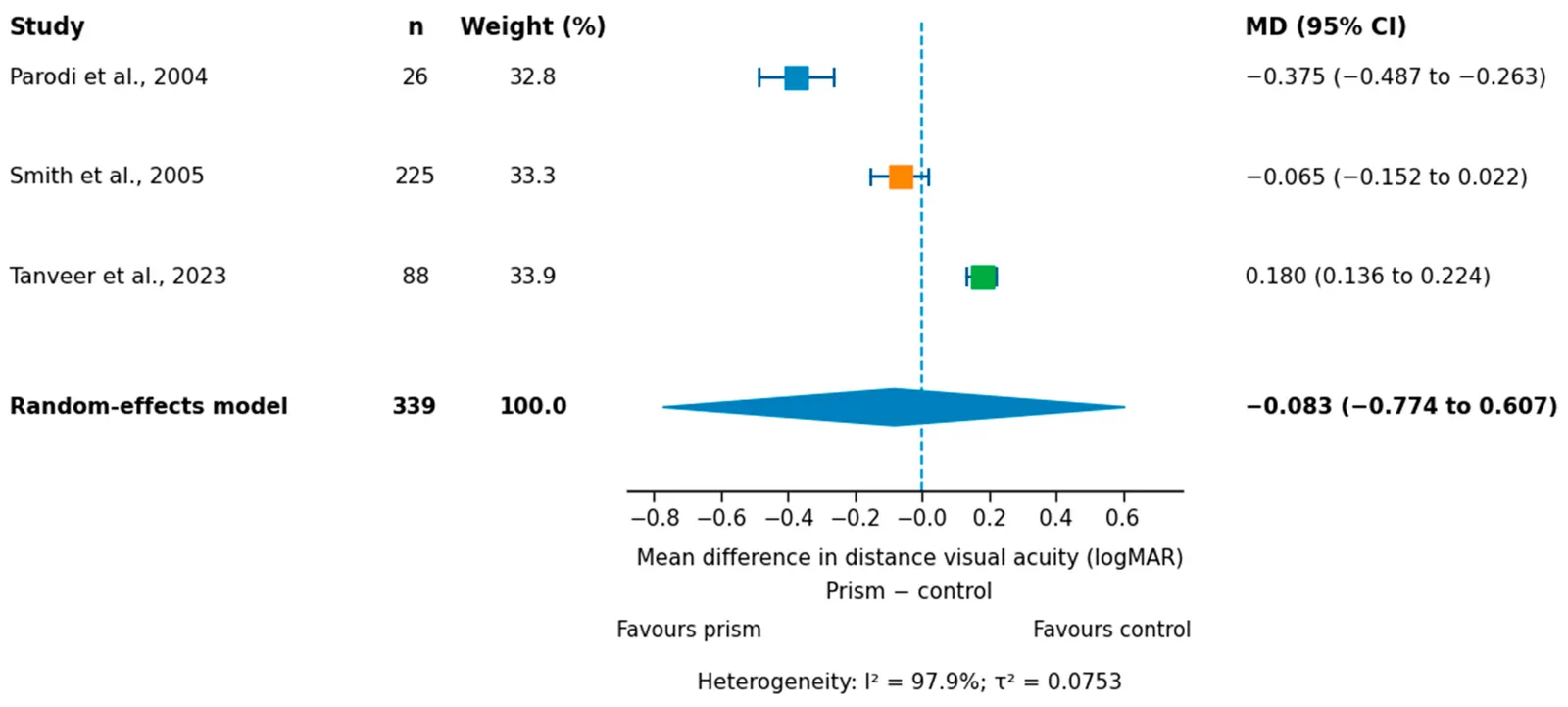
Sensitivity analysis including Tanveer et al. [23], based on the numerical distance-visual-acuity values reported in the published table. The blue square represents Parodi et al. [14], the orange square represents Smith et al. [17], and the green square represents Tanveer et al. [23]. Square sizes are proportional to study weights, and horizontal lines represent 95% confidence intervals (CIs). The blue diamond represents the pooled effect estimate, with its width indicating the Hartung–Knapp-adjusted 95% CI. Random-effects estimates were calculated using restricted maximum likelihood (REML). Negative values favour prism correction. Tanveer et al. was excluded from the primary analysis because the numerical data were inconsistent with the narrative interpretation and clarification could not be obtained.

When the published numerical values from Tanveer et al. [23] were added to the two primary trials, the REML pooled estimate moved towards the null (MD, −0.083 logMAR; Hartung–Knapp 95% CI, −0.774 to 0.607), with very substantial heterogeneity (I^2^ = 97.9%; τ^2^ = 0.0753) (Figure 3). This analysis did not strengthen evidence for prism efficacy.

**Figure 4:**
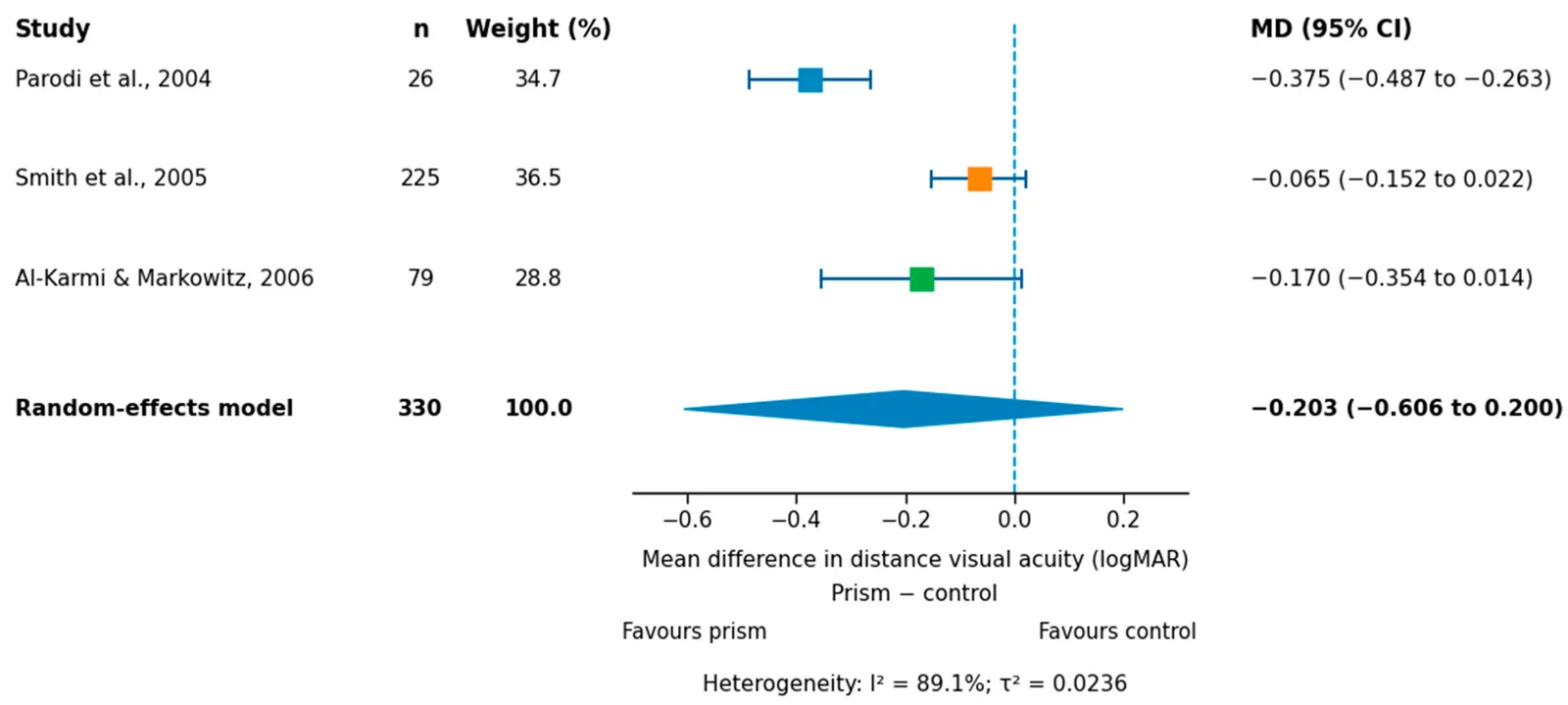
Sensitivity analysis including the non-randomized comparative study by Al-Karmi and Markowitz [12]. The blue square represents Parodi et al. [14], the orange square represents Smith et al. [17], and the green square represents Al-Karmi and Markowitz [12]. Square sizes are proportional to study weights, and horizontal lines indicate 95% confidence intervals (CIs). The blue diamond represents the pooled effect estimate, with its width indicating the Hartung–Knapp-adjusted 95% CI. Random-effects estimates were calculated using restricted maximum likelihood (REML). Negative values favour prism correction. This analysis should be interpreted cautiously because Al-Karmi and Markowitz was judged to be at critical risk of bias.

Inclusion of the non-randomized comparative study by Al-Karmi and Markowitz [12] yielded a pooled MD of −0.203 logMAR (Hartung–Knapp 95% CI, −0.606 to 0.200; I^2^ = 89.1%; τ^2^ = 0.0236) (Figure 4). Although the point estimate remained in the direction favouring prism correction, the confidence interval included the null effect and the study was at critical risk of bias because treatment allocation depended on the initial response to prism testing.

A quantitative sensitivity analysis using the adjusted ANCOVA estimates from Smith et al. [17] could not be constructed without unsupported assumptions because the two active comparisons shared a common placebo group and the covariance between adjusted estimates was not reported. However, both published adjusted comparisons were close to the null (MD, −0.02 logMAR), consistent with the interpretation that alternative handling of the Smith data does not provide stronger evidence of prism benefit.

### 3.6. Other Visual and Functional Outcomes

Outcomes other than distance visual acuity were too heterogeneous for quantitative pooling. Differences involved outcome definitions, measurement instruments, follow-up periods, study designs, prism prescriptions, and availability of comparative data. Supplementary Table S4 maps the outcomes reported by each study, their timing and measurement approach, and the reason why they were or were not suitable for quantitative synthesis.

Near-vision and reading outcomes were reported in several studies, including near visual acuity, reading speed, critical print size, single-optotype near vision, and continuous-text performance. Although favourable changes were reported in some studies, these outcomes were not sufficiently comparable to support pooled analysis. In addition, near-vision interventions frequently incorporated refractive correction or magnification, making it difficult to isolate the effect of prismatic image relocation.

Evidence concerning PRL behaviour and fixation was similarly heterogeneous. Some studies used the existing eccentric fixation locus to determine prism prescription, whereas others directly measured short-term changes in fixation position using microperimetry. These findings demonstrate that prism placement can alter retinal image position or fixation behaviour, but they do not by themselves demonstrate establishment of a stable functional PRL. Quantitative pooling was not appropriate because studies differed in prism power, retinal-coordinate definitions, measurement conditions, adaptation periods, and follow-up.

Patient-reported and functional outcomes included vision-related quality of life, activities of daily living, locomotion, autonomy, subjective benefit, and spectacle helpfulness or use. Results were inconsistent and were derived predominantly from observational or methodologically heterogeneous studies. Consequently, these outcomes were summarized narratively rather than quantitatively.

### 3.7. Tolerability and Spectacle Use

Tolerability and continued use of prism spectacles were inconsistently reported across studies. In Parodi et al. [14], 12 of 14 participants (85.7%) tolerated the prism correction, whereas two discontinued prism use within the first 10 days because of substantial difficulty while walking. In Verezen et al. [32], 23 of 38 participants reported subjective improvement after at least 3 months of eccentric-viewing spectacle use. Vettard et al. [15] reported regular use of the prescribed prism correction in 80.6% of participants and immediate improvement in visual comfort in 90.3%. In Al-Karmi and Markowitz [12], prism users reported spectacle use ranging from 3 to 48 months (mean, 8.4 ± 11.7 months).

These findings should not be interpreted as direct measures of treatment efficacy. In particular, long-term use in Al-Karmi and Markowitz was observed in a population in which prism prescription was itself conditioned by a favourable initial response to prism testing. Across studies, adverse effects, mobility-related difficulties, adherence, and acceptability were not assessed using standardized definitions or instruments. Quantitative synthesis of tolerability or continued spectacle use was therefore not appropriate.

## 4. Discussion

The present systematic review and meta-analysis provides a comprehensive synthesis of the available evidence on prismatic image relocation for visual rehabilitation in patients with macular disease and central vision loss. The literature spans more than three decades and includes randomized controlled trials, comparative observational studies, case series, and experimental within-subject designs. Despite the long-standing clinical interest in this approach, the evidence remains limited and methodologically heterogeneous.

In the primary quantitative synthesis, the pooled point estimate favoured prism correction; however, the Hartung–Knapp-adjusted confidence interval was extremely wide and clearly included the null effect. The two contributing randomized trials also showed markedly different individual effects, and substantial between-study heterogeneity was present. Accordingly, the pooled estimate should be regarded as exploratory rather than as a conventional estimate of treatment efficacy. The certainty of evidence for the primary outcome was rated as very low after downgrading for risk of bias, inconsistency, and imprecision.

Our findings provide a more cautious interpretation than some earlier reports of prism-based rehabilitation. Several observational studies described improvements in visual acuity or functional performance after image relocation [11,12,13,14,15], and a previous evidence-based review concluded that prisms could improve distance visual acuity following macular function loss [19]. However, the apparent benefit was largely derived from studies with markedly different designs and, in some cases, without an independent untreated or sham-treated comparison group. In contrast, the present review distinguished randomized evidence from uncontrolled and non-randomized evidence and restricted the primary meta-analysis to sufficiently comparable randomized studies. This distinction is important because improvements observed after low-vision intervention may result from several concurrent factors, including refractive optimization, magnification, training, repeated testing, adaptation to eccentric viewing, or selective prescription to patients who respond favourably during fitting [20,34,35].

This issue is particularly evident in the study by Al-Karmi and Markowitz [12]. Patients who perceived benefit during prism testing were subsequently prescribed prism spectacles, whereas those in whom prism correction was not considered beneficial formed the non-prism comparison group. Such treatment selection makes the groups intrinsically different with respect to initial treatment response and substantially limits causal interpretation. The more favourable results reported in this type of observational cohort should therefore not be considered equivalent to an effect demonstrated in randomized comparisons. This consideration also supports our decision to include this study only in sensitivity analysis rather than in the primary pooled estimate.

The considerable heterogeneity between the randomized trials is clinically plausible. Prism prescription differed in power, orientation, monocular or binocular application, and degree of individualization [14,17]. The trials also differed in comparator design, baseline visual status, the method used to determine prism orientation or the intended retinal target, and the extent of accompanying low-vision care. Neither trial objectively confirmed the effective retinal displacement using fundus-tracked testing. These differences provide a plausible clinical explanation for at least part of the observed heterogeneity and further limit interpretation of a single pooled treatment effect.

Patients also differed in severity of macular damage, eccentric fixation characteristics, residual visual function, and possibly duration of visual loss. More broadly, systematic reviews of low-vision rehabilitation have repeatedly emphasized substantial heterogeneity in interventions, patient characteristics, outcome measures, and service-delivery models [20,34,35]. In a randomized trial of different models of low-vision rehabilitation in AMD, Reeves et al. found no additional advantage of an enhanced home-based model over conventional rehabilitation despite high use of low-vision devices at follow-up [36]. These findings illustrate why improvement after rehabilitation cannot automatically be attributed to any single component of a multifaceted intervention.

A central rationale for prismatic image relocation is the presence of a preferred retinal locus. However, current knowledge indicates that the PRL is considerably more complex than a fixed eccentric substitute for the fovea. The PRL may be task dependent, more than one locus may be used, and fixation behaviour may evolve over time [2,3]. Longitudinal work by Crossland et al. showed that patients with recent central visual loss can develop a repeatable PRL over the months following the onset of bilateral macular disease, while some patients use multiple retinal loci [37,38]. Importantly, the naturally adopted PRL does not necessarily coincide with the retinal location providing the best visual acuity [4]. Bernard and Chung demonstrated that visual acuity could be better at retinal locations away from the habitual PRL, suggesting that PRL selection is influenced by factors beyond local resolution alone [4]. This is highly relevant to prism prescription because relocating an image toward an anatomically available retinal region does not necessarily guarantee improved functional performance.

The relationship between PRL position and visual function is further complicated by fixation stability. Fixation characteristics have been associated with reading performance in patients with macular disease, while PRL eccentricity itself does not fully explain functional ability [6,7,37]. The findings of Lewerenz et al. [18] are therefore particularly relevant: prism placement can produce measurable changes in retinal image location, but the resulting displacement is not necessarily equivalent to stable adoption of a new functional PRL. Accordingly, prism studies should distinguish explicitly between three non-equivalent outcomes: optical retinal-image displacement, sustained oculomotor adaptation or relocation of fixation, and measurable functional benefit. Demonstration of the first does not establish either of the latter two.

Microperimetry offers an important means of addressing this issue. Unlike visual acuity alone, fundus-tracked microperimetry permits simultaneous assessment of retinal sensitivity, fixation location, fixation stability, and their relationship to retinal structure [39]. Systematic evidence suggests that retinal sensitivity measured by microperimetry can detect functional abnormalities and progression in AMD that may not be fully captured by conventional visual acuity testing, although variation in testing protocols remains an important limitation [39,40]. This has direct implications for future prism trials. Demonstrating that an image has been displaced is insufficient; studies should establish whether the prism consistently redirects fixation toward a retinal area with greater sensitivity or superior task-specific function and whether this relocation persists under binocular, real-world viewing conditions.

Near-vision and reading outcomes require particular caution because these functions depend on multiple components of low-vision rehabilitation. Magnification, refractive optimization, reading training, and repeated practice can independently improve performance [41,42,43,44]. Consequently, when prism spectacles incorporate additional optical correction or rehabilitation, improvements in reading or near vision cannot be attributed specifically to retinal-image relocation.

Evidence from eccentric-viewing and microperimetric rehabilitation further illustrates this distinction. The EFFECT randomized trial found no systematic functional advantage of training a habitual or theoretically optimal retinal locus over standard low-vision rehabilitation [45], whereas smaller studies of microperimetric biofeedback have reported improvements in visual or fixation-related outcomes [46,47]. Taken together, these findings suggest that selecting or redirecting fixation toward a retinal location does not necessarily produce functional improvement and that response may depend on disease phenotype, residual retinal function, fixation stability, training intensity, and patient selection. Patient-reported functional outcomes therefore remain important complementary endpoints, particularly because changes in chart visual acuity may not correspond directly to changes in everyday visual performance [48].

The clinical implications of the present findings should therefore be considered within an individualized rehabilitation framework. Current evidence does not support the assumption that a fixed prism strategy will produce a predictable improvement across patients with central vision loss. At the same time, it does not rule out clinically relevant benefit in selected individuals. PRL position, fixation stability, scotoma morphology, residual retinal sensitivity, binocular status, visual task requirements, and adaptation capacity may all influence treatment response [2,4,6,7,39]. Contemporary low-vision rehabilitation increasingly recognizes this heterogeneity, with intervention selection based on individual goals and residual visual function rather than visual acuity alone [34,35,49]. The Veterans Affairs Low Vision Intervention Trial II, for example, showed that adding structured rehabilitation therapy to provision of low-vision devices can improve visual ability in patients with macular disease, emphasizing the value of training and task-specific rehabilitation alongside optical devices [49].

The present review has several strengths. A broad search strategy was applied across four major databases without restrictions on publication date or language, and 782 unique records were screened after deduplication. Different study designs were retained in order to characterize the complete clinical evidence base, but design-specific risk-of-bias tools were used and randomized evidence was kept separate from non-randomized evidence in the primary quantitative synthesis. Sensitivity analyses were used to evaluate the influence of studies with important methodological or reporting concerns. This approach is especially relevant in a field in which much of the historical evidence consists of small observational series and individualized clinical prescriptions.

Several limitations should be acknowledged. First, only two sufficiently comparable randomized trials contributed to the primary meta-analysis of distance visual acuity. Consequently, the pooled estimate is highly imprecise, and estimates of between-study heterogeneity are themselves unstable. Second, interventions differed in prism design, power, orientation, monocular or binocular application, fitting strategy, and associated optical or rehabilitation components. Disease phenotype, scotoma characteristics, baseline visual function, and outcome definitions also varied considerably across studies. Third, 24 of the 44 reports sought for retrieval could not be obtained. Although sufficiently informative abstracts or other available reports were considered when they permitted eligibility assessment, incomplete retrieval may have resulted in relevant studies being missed and limited the methodological assessment of some included studies. Fourth, Embase and CINAHL were not included in the prospectively registered search strategy. Although four complementary databases were searched together with backward and forward citation tracking, relevant reports indexed exclusively in databases not searched cannot be completely excluded. Fifth, outcomes other than distance visual acuity were insufficiently standardized to permit quantitative pooling. Finally, several observational studies were particularly vulnerable to selection and confounding because prism prescription depended on the initial response observed during clinical fitting. These limitations preclude a definitive estimate of the efficacy of prismatic image relocation.

Future trials should use adequately powered randomized designs with clearly described allocation procedures, appropriate masking whenever feasible, and prespecified analytical plans. Disease phenotype, scotoma morphology, residual foveal function, baseline PRL characteristics, and binocular status, including ocular dominance, suppression, and stereopsis when measurable, should be characterized systematically. Prism prescription should report power, base direction, monocular or binocular application, degree of individualization, and the intended retinal target. Importantly, prism power should not be assumed to represent effective retinal displacement; fundus-tracked microperimetry should be used whenever possible to document the actual change in retinal image position, fixation location and stability, and retinal sensitivity before and after an appropriate adaptation period. Visual acuity should be assessed using standardized logMAR procedures, but future trials should also include reading performance, task-based visual function, patient-reported outcomes, adherence, mobility, comfort, and adverse effects. Determining whether specific baseline structural or functional characteristics predict clinically meaningful response may ultimately be more informative than estimating a single average effect across a highly heterogeneous population.

## 5. Conclusions

Current evidence is insufficient to establish the routine efficacy of prismatic image relocation for improving distance visual acuity in patients with macular disease and central vision loss. Although prism correction can alter retinal image position and may provide visual or functional benefit in selected individuals, the randomized evidence is limited, highly heterogeneous, and of very low certainty. The pooled estimate from the available randomized trials should therefore be regarded as exploratory rather than as evidence of an established treatment effect.

An individualized trial of prism correction may nevertheless be reasonable in selected patients within structured low-vision rehabilitation, particularly when the intervention is tailored to residual retinal function and specific visual goals. Future adequately powered randomized trials should incorporate standardized visual and functional outcomes, objective fundus-tracked assessment of retinal image displacement and fixation behaviour, characterization of binocular status and disease phenotype, and prospective evaluation of adherence, tolerability, and adverse effects. Identifying which patients are most likely to achieve clinically meaningful benefit may ultimately be more informative than estimating a single average treatment effect across a heterogeneous population.

## Figures and Tables

**Table 1:** Risk-of-bias and methodological quality assessment of the included studies.

Study	Study Design	Assessment Tool	Overall Judgment *	Main Methodological Concerns
Rosenberg et al., 1989 [24]	Quasi-randomized comparative trial	ROBINS-I	Serious risk	Predictable allocation based on odd/even case number
Verezen et al., 1996 [32]	Observational follow-up/case series	JBI	—	Consecutive and complete inclusion and demographic reporting unclear
Bertrand et al., 1997 [33]	Case series	JBI	—	Consecutive and complete inclusion and demographic reporting unclear
Parodi et al., 2004 [14]	Randomized controlled trial	RoB 2	Some concerns	Limited reporting of allocation/masking and post-randomization discontinuations
Vettard et al., 2004 [15]	Treatment cohort/case series	JBI	—	Consecutive and complete inclusion and demographic reporting unclear
Smith et al., 2005 [17]	Randomized, double-masked, placebo-controlled trial	RoB 2	Some concerns	Missing outcome data
Al-Karmi & Markowitz, 2006 [12]	Non-randomized comparative study	ROBINS-I	Critical risk	Confounding and participant selection; treatment assignment depended on initial prism response
Reyes et al., 2013 [13]	Prospective non-randomized study	JBI	—	No comparable concurrent control group; co-interventions and follow-up incompletely reported
Lewerenz et al., 2018 [18]	Within-subject pilot study	JBI	—	No independent parallel control group; within-participant comparison design
Markowitz et al., 2021 [11]	Observational/pre-post case series	JBI	—	No concurrent control group
Tanveer et al., 2023 [23]	Randomized controlled trial	RoB 2	High risk	Concerns regarding randomization and selective reporting; internal inconsistency between numerical and narrative results

Note: RoB 2 was used for genuinely randomized controlled trials and ROBINS-I for quasi-randomized and non-randomized comparative studies. JBI assessments are reported at the individual-item level; no synthetic overall JBI category was assigned. Detailed assessments are provided in Supplementary Tables S6–S8. * Overall judgment applies only to studies assessed using RoB 2 or ROBINS-I; no overall judgment was assigned to studies assessed using JBI checklists.

**Table 2:** Clinical and methodological characteristics of the randomized trials contributing to the primary meta-analysis.

Characteristic	Parodi et al., 2004 [14]	Smith et al., 2005 [17]
Study design	Randomized controlled clinical trial	Double-masked, randomized placebo-controlled trial
Population	Advanced bilateral exudative AMD; VA > 0.5 logMAR and stable ≥ 1 year	Bilateral AMD with low vision; better-eye VA 0.48–1.78 logMAR
Randomized sample	28 (14 prism; 14 control)	243 randomized
Participants assessed at ≈3 months	26	225
Baseline distance VA	Prism: 1.06 ± 0.17 logMAR; control: 1.06 ± 0.21 logMAR	Custom: 0.88 ± 0.33; standard: 0.89 ± 0.35; placebo: 0.94 ± 0.31 logMAR
Prism prescription	Monocular prism in the better eye, 4–7Δ	Bilateral custom prisms or standard bilateral prisms (6Δ or 10Δ)
Prism orientation	Prism rotated to the position providing clearest vision	Custom: preferred power and base direction; standard: predefined base-up prescription
Individualization	Individualized orientation	Custom arm individualized; standard arm predefined
Comparator	Best optical correction without prism	Placebo spectacles matched for weight and thickness, including optimal refractive correction
Concomitant low-vision rehabilitation	No structured additional rehabilitation reported as part of the intervention	Standard low-vision assessment and conventional low-vision aids provided before the study intervention
PRL/retinal target assessment	Functional clearest-vision position; no fundus-tracked confirmation reported	Preferred prism orientation determined subjectively; no fundus-tracked confirmation reported
Distance VA assessment	ETDRS, logMAR	ETDRS, logMAR; binocular
Follow-up used in synthesis	90 days	3 months
Published primary VA result	Significant improvement reported in the prism group versus control	No significant treatment effect after baseline adjustment (overall *p* = 0.63)

Note: AMD, age-related macular degeneration; ETDRS, Early Treatment Diabetic Retinopathy Study; PRL, preferred retinal locus; VA, visual acuity; Δ, prism diopters.

## Data Availability

The data supporting the findings of this systematic review and meta-analysis were derived from previously published studies and are reported in the article and its Supplementary Materials. The extracted data used for the quantitative analyses and the risk-of-bias and certainty-of-evidence assessments are available from the corresponding author upon reasonable request.

## Supplementary Materials

The following supporting information can be downloaded at: https://www.mdpi.com/article/10.3390/jcm15218322/s1, Table S1: PRISMA 2020 checklist; Table S2: Database-specific adaptations of the search strategy used for study identification; Table S3: GRADE assessment of the certainty of evidence for the primary outcome; Table S4: Outcome availability and suitability for quantitative synthesis across included studies; Table S5: Characteristics of the studies included in the systematic review; Table S6: RoB 2 assessment of genuinely randomized controlled trials; Table S7: ROBINS-I assessment of quasi-randomized and non-randomized comparative studies included in the systematic review; Table S8: JBI critical appraisal of observational and quasi-experimental studies included in the systematic review.

## Abbreviations

The following abbreviations are used in this manuscript:

Abbreviation	Definition
AMD	Age-related macular degeneration
CI	Confidence interval
CENTRAL	Cochrane Central Register of Controlled Trials
GRADE	Grading of Recommendations Assessment, Development and Evaluation
JBI	Joanna Briggs Institute
logMAR	Logarithm of the minimum angle of resolution
MD	Mean difference
PRISMA	Preferred Reporting Items for Systematic Reviews and Meta-Analyses
PRL	Preferred retinal locus
RoB 2	Risk of Bias 2
ROBINS-I	Risk Of Bias In Non-randomized Studies of Interventions
RR	Risk ratio
SMD	Standardized mean difference
