# Supplementary material for: Prismatic Image Relocation in Macular Disease: Effects on Visual Function and the Preferred Retinal Locus—A Systematic Review and Meta-Analysis
Source: J Clin Med. 2026 Oct 26;15(21):8322. doi: 10.3390/jcm15218322 (PMC13764528; doi:10.3390/jcm15218322)
Supplement: Supplementary file 1 [file jcm-15-08322-s001.zip › Supplementary_Table S1_PRISMA.pdf]

Supplementary Table S1. PRISMA 2020 Checklist

Manuscript title: Prismatic Image Relocation in Macular Disease: Effects on Visual Function and the Preferred Retinal Locus—A Systematic Review and Meta-Analysis

PROSPERO registration: CRD420261500738

This checklist maps the PRISMA 2020 reporting items to the current manuscript and supplementary material. Locations are given by section and supplement rather than fixed page numbers so that the checklist remains valid after journal formatting.

PRISMA item	Topic	Checklist detail / how addressed	Location in manuscript or supplement
1	Title	Identify the report as a systematic review and meta-analysis.	Title page
2	Abstract	Provide a structured summary of the review objectives, methods, principal results, certainty of evidence, and conclusions.	Abstract
3	Rationale	Explain the rationale for evaluating prism-based image relocation in macular disease and the limitations of the existing evidence.	Introduction, paragraphs 1–5
4	Objectives	State the objective of evaluating visual acuity, reading-related outcomes, fixation/PRL behaviour, and patient-reported functional outcomes.	End of Introduction
5	Eligibility criteria	Specify eligible populations, prism-based interventions, study designs, comparators, and exclusion criteria.	Methods 2.3 Eligibility criteria
6	Information sources	List Scopus, Web of Science Core Collection, PubMed/MEDLINE, CENTRAL, and reference-list checking; state that searches covered each database from inception and were conducted between 2 and 15 September 2026.	Methods 2.2 Information sources and search strategy; Supplementary Table S2
7	Search strategy	Provide the core search structure and database-specific adaptations and searchable fields; report the complete database-specific strategies and records retrieved.	Methods 2.2 Information sources and search strategy; Supplementary Table S2
8	Selection process	Describe independent two-reviewer title/abstract screening, Include/Exclude/Uncertain classification, third-reviewer adjudication, full-text assessment, and handling of sufficiently detailed abstracts.	Methods 2.4 Study selection; Figure 1 and caption
9	Data collection process	Describe independent duplicate extraction, standardized extraction fields, consensus procedures, and use of full text or sufficiently detailed abstracts.	Methods 2.5 Data extraction
10a	Data items – outcomes	Define the primary outcome (distance visual acuity) and secondary outcomes including near vision, reading, contrast sensitivity, fixation/PRL, microperimetry, quality of life, tolerability, adherence, and improvement proportions.	Methods 2.8 Outcomes
10b	Data items – other variables	Record study design, population, sample size, diagnosis, prism characteristics, PRL identification method, comparator, follow-up, and measures of precision.	Methods 2.5 Data extraction; Supplementary Table S5
11	Study risk of bias assessment	Describe independent risk-of-bias assessment using RoB 2, ROBINS-I, and design-appropriate JBI tools, including resolution of disagreements and treatment of abstract-only reports.	Methods 2.6 Risk of bias assessment; Results 3.3; Table 1; Supplementary Tables S6–S8
12	Effect measures	Specify mean differences with 95% CIs for continuous outcomes on the same scale, standardized mean differences when appropriate, and risk ratios for dichotomous outcomes.	Methods 2.9 Statistical analysis
13a	Synthesis methods – study eligibility	Explain that meta-analysis was undertaken when at least two studies were sufficiently comparable and that controlled and uncontrolled studies were handled separately.	Methods 2.9 Statistical analysis
13b	Synthesis methods – data preparation	Describe preference for baseline-adjusted estimates when directly comparable, use of change scores when appropriate, final values otherwise, and conversion to logMAR when methodologically appropriate.	Methods 2.8 Outcomes
13c	Synthesis methods – tabulation/visual display	Present PRISMA flow, risk-of-bias summary, primary forest plot, sensitivity-analysis forest plots, and detailed supplementary study/risk-of-bias tables.	Figure 1; Table 1; Figures 2–4; Supplementary Tables S4–S8
13d	Synthesis methods – statistical methods	Describe random-effects meta-analysis using REML for τ^2 and Hartung–Knapp-adjusted 95% CIs; conventional REML and DerSimonian–Laird analyses were examined in sensitivity analyses, with narrative synthesis when pooling was inappropriate.	Methods 2.9 Statistical analysis
13e	Synthesis methods – heterogeneity	Assess heterogeneity using τ^2, I^2, and Cochran's Q and interpret heterogeneity descriptively because of the small number of contributing studies.	Methods 2.9 Statistical analysis
13f	Synthesis methods – sensitivity analyses	Describe sensitivity analyses of statistical method and alternative study/effect-estimate inclusion, including Smith et al. (2005), Tanveer et al. (2023), and Al-Karmi and Markowitz (2006).	Methods 2.9 Statistical analysis; Results 3.5; Figures 3–4
14	Reporting bias assessment	State that funnel plots and asymmetry tests were planned only when at least 10 studies contributed to the same meta-analysis; this threshold was not met.	Methods 2.7 Reporting bias and certainty of evidence
15	Certainty assessment	Describe GRADE assessment of the primary outcome across risk of bias, inconsistency, indirectness, imprecision, and publication bias.	Methods 2.7 Reporting bias and certainty of evidence; Results 3.4; Supplementary Table S3
16a	Study selection results	Report 1167 identified records, 385 duplicates removed, 782 screened, 738 excluded at title/abstract, 44 reports sought, 24 not retrieved, 20 assessed, 9 excluded, and 11 included studies.	Results 3.1; Figure 1
16b	Excluded studies	Report full-text exclusion categories and counts and cite the excluded reports assessed at eligibility stage.	Results 3.1; Figure 1 and caption
17	Study characteristics	Summarize design, diagnosis, sample, intervention, comparator, follow-up, and outcomes for included studies.	Results 3.2; Supplementary Table S5

PRISMA item	Topic	Checklist detail / how addressed	Location in manuscript or supplement
18	Risk of bias in studies	Report study-level risk-of-bias and methodological-quality judgments, with detailed domain/item-level assessments in the supplement.	Results 3.3; Table 1; Supplementary Tables S6–S8
19	Results of individual studies	Report study-level visual, functional, PRL/fixation, patient-reported, and tolerability findings; detailed characteristics and outcome availability are provided in S1 and S8.	Results 3.4–3.7; Supplementary Tables S5 and S4
20a	Results of syntheses – contributing studies	Identify the two randomized trials contributing to the primary meta-analysis and distinguish them from studies used in sensitivity or narrative analyses.	Results 3.4–3.5; Table 2; Figures 2–4
20b	Results of syntheses – statistical results	Report the primary pooled MD (−0.218 logMAR; Hartung–Knapp 95% CI, −2.188 to 1.753), I^2 = 94.5%, τ^2 = 0.0455, and numerical sensitivity-analysis estimates.	Results 3.4–3.5; Figures 2–4
20c	Results of syntheses – heterogeneity	Report substantial heterogeneity and interpret it in relation to differences in prism prescription, populations, and study methods.	Results 3.4–3.5; Discussion
20d	Results of syntheses – sensitivity analyses	Report sensitivity analyses including Tanveer et al. (2023) and Al-Karmi and Markowitz (2006), and explain why neither was included in the primary pooled estimate.	Results 3.5; Figures 3–4
21	Reporting biases	Explain that formal small-study/publication-bias testing was not performed because fewer than 10 studies contributed to the meta-analysis.	Methods 2.7; limitation implicit in quantitative synthesis
22	Certainty of evidence	Report very low certainty for the primary outcome after downgrading one level each for risk of bias, inconsistency, and imprecision.	Results 3.4; Discussion opening; Conclusions; Supplementary Table S3
23a	Discussion – interpretation	Interpret the pooled estimate in the context of observational evidence, PRL biology, microperimetry, reading rehabilitation, and eccentric-viewing training.	Discussion
23b	Discussion – limitations of evidence	Discuss the small number of randomized trials, extreme imprecision, intervention and disease heterogeneity, variable outcomes, and bias in observational evidence.	Discussion, limitations paragraph
23c	Discussion – limitations of review processes	Discuss the 24 reports not retrieved, use of abstract/incomplete reports when sufficiently informative, omission of Embase and CINAHL from the registered search, inability to pool most secondary outcomes, and limited power to assess reporting bias.	Discussion, limitations paragraph
23d	Discussion – implications	Describe individualized clinical use and priorities for future randomized trials incorporating microperimetry, reading, patient-reported outcomes, adherence, mobility, and adverse effects.	Discussion, clinical implications and future-trials paragraphs; Conclusions
24a	Registration	Report prospective PROSPERO registration CRD420261500738.	Abstract; Methods 2.1 Study design and protocol
24b	Protocol availability	Identify the PROSPERO registration record as the protocol source.	Methods 2.1 Study design and protocol
24c	Protocol amendments	Report two post-registration analytical decisions: reclassification of Rosenberg et al. as quasi-randomized and exclusion of Tanveer et al. from the primary meta-analysis because of an unresolved internal reporting inconsistency. A single procedural deviation occurred during peer review: following a reviewer request, an attempt was made to contact the authors of Tanveer et al. for clarification, despite the registered protocol stating that study authors would not be contacted for additional information.	Methods 2.10 Protocol deviations and post-hoc analytical decisions
25	Support	Report funding source(s) and the role of funders or sponsors.	The review received no external funding.
26	Competing interests	Report authors' competing interests.	The authors declare no conflicts of interest.
27	Availability of data, code and materials	Describe availability of the extracted dataset, risk-of-bias assessments, and supplementary materials.	Data Availability Statement; Supplementary Tables S2–S8; Supplementary File S1
