# Supplementary material for: Prismatic Image Relocation in Macular Disease: Effects on Visual Function and the Preferred Retinal Locus—A Systematic Review and Meta-Analysis
Source: J Clin Med. 2026 Oct 26;15(21):8322. doi: 10.3390/jcm15218322 (PMC13764528; doi:10.3390/jcm15218322)
Supplement: Supplementary file 1 [file jcm-15-08322-s001.zip › Supplementary_Table_S2 (2).pdf]

Supplementary Table S2. Database-specific adaptations of the search strategy used for study identification.

Database	Database-specific field / implementation	Search strategy	Search period	Records retrieved
Scopus	TITLE-ABS-KEY	TITLE-ABS-KEY(("prism*" OR "prismatic correction" OR "prism spectacle*" OR "yoked prism*" OR "Fresnel prism*" OR "image relocation" OR "relocating prism*") AND ("macular degeneration" OR "age-related macular degeneration" OR AMD OR "macular disease*" OR maculopath* OR "macular dystroph*" OR Stargardt OR "central scotoma*" OR "central vision loss" OR "preferred retinal locus" OR PRL OR "eccentric fixation" OR "eccentric viewing"))	2–15 Sep 2026	705
Web of Science Core Collection	Topic search (TS)	TS=(("prism*" OR "prismatic correction" OR "prism spectacle*" OR "yoked prism*" OR "Fresnel prism*" OR "image relocation" OR "relocating prism*") AND ("macular degeneration" OR "age-related macular degeneration" OR AMD OR "macular disease*" OR maculopath* OR "macular dystroph*" OR Stargardt OR "central scotoma*" OR "central vision loss" OR "preferred retinal locus" OR PRL OR "eccentric fixation" OR "eccentric viewing"))	2–15 Sep 2026	224
PubMed/MEDLINE	Equivalent Boolean combination of the same concepts using the PubMed/MEDLINE searchable fields.	("prism*" OR "prismatic correction" OR "prism spectacle*" OR "yoked prism*" OR "Fresnel prism*" OR "image relocation" OR "relocating prism*") AND ("macular degeneration" OR "age-related macular degeneration" OR AMD OR "macular disease*" OR maculopath* OR "macular dystroph*" OR Stargardt OR "central scotoma*" OR "central vision loss" OR "preferred retinal locus" OR PRL OR "eccentric fixation" OR "eccentric viewing")	2–15 Sep 2026	190
CENTRAL	Equivalent Boolean combination of the same concepts adapted to the CENTRAL search interface.	("prism*" OR "prismatic correction" OR "prism spectacle*" OR "yoked prism*" OR "Fresnel prism*" OR "image relocation" OR "relocating prism*") AND ("macular degeneration" OR "age-related macular degeneration" OR AMD OR "macular disease*" OR maculopath* OR "macular dystroph*" OR Stargardt OR "central scotoma*" OR "central vision loss" OR "preferred retinal locus" OR PRL OR "eccentric fixation" OR "eccentric viewing")	2–15 Sep 2026	48

Note: Searches across the four databases were executed on different dates between 2 and 15 September 2026. The same conceptual Boolean structure was used across databases and adapted to the syntax and searchable fields supported by each platform. No publication-date or language restrictions were applied. Exact individual execution dates were not separately archived.
