# Supplementary material for: Prismatic Image Relocation in Macular Disease: Effects on Visual Function and the Preferred Retinal Locus—A Systematic Review and Meta-Analysis
Source: J Clin Med. 2026 Oct 26;15(21):8322. doi: 10.3390/jcm15218322 (PMC13764528; doi:10.3390/jcm15218322)
Supplement: Supplementary file 1 [file jcm-15-08322-s001.zip › Supplementary_Table_S3 (2).pdf]

Supplementary Table S3. GRADE assessment of the certainty of evidence for the primary outcome.

Outcome	Studies	Participants	Effect estimate	Risk of bias	Inconsistency	Indirectness	Imprecision	Publication bias	Certainty
Distance visual acuity (logMAR), approximately 3 months	2 RCTs	251*	REML MD −0.218 logMAR (Hartung–Knapp 95% CI −2.188 to 1.753)	Serious (−1)[1]	Serious (−1)[2]	Not serious (0)	Serious (−1)[3]	Not downgraded[4]	Very low

Abbreviations: CI, confidence interval; MD, mean difference; RCT, randomized controlled trial; REML, restricted maximum likelihood.

*Participants contributing to the comparative endpoint data used in the primary meta-analysis: Parodi et al. (n = 26) and Smith et al. (n = 225), total n = 251.

GRADE explanations:

[1] Risk of bias: Downgraded one level because both randomized trials were judged as having some concerns. In Parodi et al., post-randomization discontinuations and limited information on allocation and masking were relevant concerns, while Smith et al. had some concerns related mainly to missing outcome data.

[2] Inconsistency: Downgraded one level because the magnitude of the treatment effect differed markedly between the two trials, with substantial statistical heterogeneity (I^2 = 94.5%; REML τ^2 = 0.0455). Given that only two studies contributed to the meta-analysis, heterogeneity estimates remain imprecise, but the difference in study-specific effects is clinically evident.

[3] Imprecision: Downgraded one level because the Hartung–Knapp confidence interval was extremely wide and crossed the null effect, encompassing both potentially important benefit and possible harm. This reflects the uncertainty associated with a random-effects synthesis based on only two trials.

[4] Publication bias: Not downgraded. Publication bias could not be formally assessed because only two studies contributed to the meta-analysis.
