# Supplementary material for: Prismatic Image Relocation in Macular Disease: Effects on Visual Function and the Preferred Retinal Locus—A Systematic Review and Meta-Analysis
Source: J Clin Med. 2026 Oct 26;15(21):8322. doi: 10.3390/jcm15218322 (PMC13764528; doi:10.3390/jcm15218322)
Supplement: Supplementary file 1 [file jcm-15-08322-s001.zip › Supplementary_Table_S4 (2).pdf]

**Supplementary Table S4.** Outcome availability and suitability for quantitative synthesis across included studies.

| Study | Distance VA | Near vision / reading | PRL / fixation / microperimetry | Contrast / other visual measures | Patient-reported / functional outcomes | Spectacle use / tolerability | Follow-up | Quantitative synthesis status |
|---|---|---|---|---|---|---|---|---|
| Rosenberg et al., 1989 | Yes | No | No quantitative PRL outcome reported | No | Visual function, locomotion, subjective benefit | Not separately standardized | ≈3 months | Not pooled: quasi-randomized study and limited comparable numerical data. |
| Verezen et al., 1996 | No primary quantitative VA outcome reported | No | PRL used to fit eccentric-viewing spectacles; no quantitative PRL outcome | No | Subjective perceived improvement | Everyday use | ≥3 months | Not pooled: uncontrolled observational study; predominantly subjective outcomes. |
| Bertrand et al., 1997 | Yes, binocular VA | No | Eccentric fixation used for prescription; no standardized PRL outcome | Contrast and colour vision | Autonomy | No standardized use/tolerability outcome | Not reported | Not pooled: case series; heterogeneous and incompletely reported outcomes. |
| Parodi et al., 2004 | Yes, ETDRS logMAR | No | No fundus-tracked PRL outcome | No | No standardized patient-reported functional scale | Tolerance/adverse effects | 1, 90, 180, 360 days | Primary meta-analysis: 90-day distance VA. |
| Vettard et al., 2004 | Yes | Yes: near VA and reading | Eccentric viewing used for prescription; qualitative stabilization reported | Contrast sensitivity | Visual comfort, autonomy | Regular use / comfort | 3–6 months | Not pooled: observational design and heterogeneous outcome definitions. |

| Study | Distance VA | Near vision / reading | PRL / fixation / microperimetry | Contrast / other visual measures | Patient-reported / functional outcomes | Spectacle use / tolerability | Follow-up | Quantitative synthesis status |
|---|---|---|---|---|---|---|---|---|
| Smith et al., 2005 | Yes, ETDRS logMAR | Yes: reading speed and critical print size | No fundus-tracked PRL outcome | No | NEI-VFQ-25; Melbourne Low Vision ADL | Manchester Low Vision Questionnaire included helpfulness/us e | 3 months | Primary meta-analysis: 3-month distance VA. |
| Al-Karmi & Markowitz, 2006 | Yes, ETDRS logMAR | Yes | PRL location | No | No standardized patient-reported functional scale | Spectacle use | 3–48 months; mean 8.4±11.7 months | Sensitivity analysis only: non-randomized comparative study at critical risk of bias. |
| Reyes et al., 2013 | No treatment VA outcome suitable for pooling | No | Fixation stability and PRL eccentricity | No | No | No | 3 and 12 months | Not pooled: fixation/PRL outcomes not comparable with other studies and non-randomized design. |
| Lewerenz et al., 2018 | No | No | Vertical and horizontal PRL displacement by microperimetry | No | No | No | Immediat e assessme nt | Not pooled: unique immediate within-subject PRL outcome. |
| Markowitz et al., 2021 | Yes, ETDRS logMAR | No | PRL and potential visual acuity | Contrast | No | No | Post-relocation assessme nt | Not pooled: uncontrolled pre-post case series. |

| Study | Distance VA | Near vision / reading | PRL / fixation / microperimetry | Contrast / other visual measures | Patient-reported / functional outcomes | Spectacle use / tolerability | Follow-up | Quantitative synthesis status |
|---|---|---|---|---|---|---|---|---|
| Tanveer et al., 2023 | Yes, logMAR | Yes: near vision and continuous text | No | No | VFQ-25 | No standardized use/tolerability outcome | 1 week and 1 month | Sensitivity analysis only: internal inconsistency in published distance-VA reporting; follow-up not comparable with primary 3-month endpoint. |

**Abbreviations:** ADL, activities of daily living; ETDRS, Early Treatment Diabetic Retinopathy Study; PRL, preferred retinal locus; VA, visual acuity; VFQ-25, 25-item Visual Function Questionnaire.

**Note:** “Not pooled” indicates that quantitative synthesis was not considered appropriate because of study design, outcome heterogeneity, timing, incomplete reporting, or lack of sufficiently comparable studies. Tanveer et al. was retained only in sensitivity analysis because clarification of the internal reporting inconsistency could not be obtained.
