# Supplementary material for: Prismatic Image Relocation in Macular Disease: Effects on Visual Function and the Preferred Retinal Locus—A Systematic Review and Meta-Analysis
Source: J Clin Med. 2026 Oct 26;15(21):8322. doi: 10.3390/jcm15218322 (PMC13764528; doi:10.3390/jcm15218322)
Supplement: Supplementary file 1 [file jcm-15-08322-s001.zip › Supplementary_Table_S5 (2).pdf]

Supplementary Table S5. Characteristics of the studies included in the systematic review.

Study	Design	Population	Sample size	Prism intervention	Comparator	Follow-up	Main outcomes
Rosenberg et al., 1989	Quasi-randomized comparative trial; allocation by odd/even case number [FR]	Macular dysfunction and low vision	30 (19 prism; 11 control)	Prism spectacles; power and axis individualized after trial fitting	Similar spectacles without prism	≈3 months	Visual acuity, visual function, locomotion, subjective benefit
Verezen et al., 1996	Observational follow-up study [IR]	End-stage AMD with central scotoma	38	High-power eccentric-viewing prism spectacles fitted to the PRL	None	≥3 months	Subjective perceived improvement / everyday use
Bertrand et al., 1997	Clinical case series [IR]	Bilateral macular disease; AMD	14	Bilateral prism correction based on eccentric fixation	None	Not reported	Binocular visual acuity, contrast, colour vision, autonomy
Parodi et al., 2004	Randomized controlled clinical trial [FR]	Advanced bilateral exudative AMD; VA >0.5 logMAR and stable ≥1 year	28 randomized (14 prism; 14 control); 12 prism patients at later follow-up	Monocular 4–7Δ prism before the better eye; rotated to clearest-vision position	Best optical correction without prism	1, 90, 180 and 360 days	Distance visual acuity (ETDRS, logMAR), tolerance/adverse effects

Vettard et al., 2004	Treatment cohort [IR]	AMD with bilateral central scotomas	31	Bilateral prisms oriented according to eccentric fixation	None	3–6 months	Distance/near visual acuity, reading, contrast, comfort, use, autonomy
Smith et al., 2005	Double-masked, placebo-controlled randomized trial [FR]	AMD with low vision	243 randomized; 225 assessed at 3 months	Custom bilateral prisms or standard bilateral prisms (6Δ or 10Δ base up)	Placebo spectacles matched for weight/thickness	3 months	Distance visual acuity, reading speed, critical print size, NEI-VFQ-25, Melbourne Low Vision ADL
Al-Karmi & Markowitz, 2006	Retrospective comparative interventional case series [FR]	AMD with central vision loss	100 (64 prism; 15 non-prism spectacles; 21 no distance prescription)	Yoked prisms for image relocation to the PRL; 2–10Δ (mean 5.8±1.9Δ)	Distance spectacles without prism / no distance prescription	Prism use 3–48 months; mean 8.4±11.7 months	BCVA (ETDRS, logMAR), near vision, PRL, spectacle use
Reyes et al., 2013	Prospective non-randomized observational study [IR]	AMD; BCVA 20/50–20/400	24 (14 AMD prism users; 10 controls without retinal pathology)	Distance spectacles with prisms for image relocation; 6±2Δ	Participants without retinal pathology for fixation comparison	3 and 12 months	Fixation stability, PRL eccentricity
Lewerenz et al., 2018	Within-subject pilot study [IR]	Central scotoma	13 participants (up to 26 eyes/cases)	6Δ and 10Δ base-up prisms; PRL displacement measured by microperimetry	No lens and non-prism control lens	Immediate assessment	Vertical and horizontal PRL displacement by microperimetry
Markowitz et al., 2021	Retrospective non-randomized observational case series [FR]	Bilateral AMD; better-eye BCVA 20/50–20/400	33	Yoked prisms directed toward the PRL; mean 5.16±1.44Δ	Pre-intervention status; no independent control group	Post-relocation assessment	BCVA (ETDRS, logMAR), potential visual acuity, contrast, PRL

Tanveer et al., 2023	Randomized controlled trial [FR]	AMD, age ≥50 years	88 (44 prism; 44 control)	Individualized bilateral prism spectacles, 6–10Δ	Similar weight/thickness spectacles without prism	1 week and 1 month	Distance BCVA (logMAR), near vision, continuous text, VFQ-25

Abbreviations: AMD, age-related macular degeneration; BCVA, best-corrected visual acuity; ETDRS, Early Treatment Diabetic Retinopathy Study; PRL, preferred retinal locus; VFQ-25, 25-item Visual Function Questionnaire; ADL, activities of daily living; Δ, prism diopters; FR, full report available for data extraction; IR, abstract/incomplete report used for data extraction and appraisal.
