# Supplementary material for: Prismatic Image Relocation in Macular Disease: Effects on Visual Function and the Preferred Retinal Locus—A Systematic Review and Meta-Analysis
Source: J Clin Med. 2026 Oct 26;15(21):8322. doi: 10.3390/jcm15218322 (PMC13764528; doi:10.3390/jcm15218322)
Supplement: Supplementary file 1 [file jcm-15-08322-s001.zip › Supplementary_Table_S6 (2).pdf]

Supplementary Table S6. RoB 2 assessment of genuinely randomized controlled trials.

Study	D1	D2	D3	D4	D5	Overall
Parodi et al., 2004	Some concerns	Some concerns	Some concerns	Low risk	Low risk	Some concerns
Smith et al., 2005	Low risk	Low risk	Some concerns	Low risk	Low risk	Some concerns
Tanveer et al., 2023	High risk	Some concerns	Some concerns	Some concerns	High risk	High risk
