# Supplementary material for: Prismatic Image Relocation in Macular Disease: Effects on Visual Function and the Preferred Retinal Locus—A Systematic Review and Meta-Analysis
Source: J Clin Med. 2026 Oct 26;15(21):8322. doi: 10.3390/jcm15218322 (PMC13764528; doi:10.3390/jcm15218322)
Supplement: Supplementary file 1 [file jcm-15-08322-s001.zip › Supplementary_Table_S7 (2).pdf]

Supplementary Table S7. ROBINS-I assessment of quasi-randomized and non-randomized comparative studies included in the systematic review.

Study	D1 Confounding	D2 Selection of participants	D3 Classification of interventions	D4 Deviations from intended interventions	D5 Missing data	D6 Measurement of outcomes	D7 Selection of reported result	Overall
Rosenberg et al., 1989	Serious	Moderate	Low	Moderate	No information	Moderate	No information	Serious
Al-Karmi & Markowitz, 2006	Critical	Critical	Low	Moderate	Moderate	Moderate	Moderate	Critical

Rosenberg et al. (1989): The study used a predictable odd/even case-number allocation procedure and was therefore treated as quasi-randomized rather than genuinely randomized. The main concerns relate to potential confounding and selection arising from the non-concealed allocation process, limited information on deviations and missing data, and incomplete reporting of prespecified analyses. Intervention classification was considered clear, whereas outcome measurement was judged at moderate risk because objective visual-acuity measures were combined with functional and subjective outcomes without sufficient information on masking.

Al-Karmi & Markowitz (2006): Critical risk of bias was primarily driven by confounding and participant selection. The study was a retrospective comparative interventional case series, and treatment allocation was determined by the observed response to prism testing rather than by an intervention assignment independent of prognosis or treatment response. Patients in whom benefit was identified by both the patient and clinician were prescribed prism spectacles, whereas patients in whom prisms were not considered beneficial constituted the non-prism comparison group. Consequently, the intervention and comparison groups were inherently selected according to their initial response to the intervention. Outcome measurement was based on a standardized clinical protocol, including ETDRS assessment of BCVA, but the retrospective design, variable follow-up duration, and lack of adjustment for potential confounding factors further limited causal interpretation.
