# Supplementary material for: Prismatic Image Relocation in Macular Disease: Effects on Visual Function and the Preferred Retinal Locus—A Systematic Review and Meta-Analysis
Source: J Clin Med. 2026 Oct 26;15(21):8322. doi: 10.3390/jcm15218322 (PMC13764528; doi:10.3390/jcm15218322)
Supplement: Supplementary file 1 [file jcm-15-08322-s001.zip › Supplementary_Table_S8 (2).pdf]

**Supplementary Table S8.** JBI critical appraisal of observational and quasi-experimental studies included in the systematic review.

**A.** JBI Critical Appraisal Checklist for Quasi-Experimental Studies

| Study | Q1 | Q2 | Q3 | Q4 | Q5 | Q6 | Q7 | Q8 | Q9 |
|---|---|---|---|---|---|---|---|---|---|
| Reyes et al., 2013 | Y | Y | U | N | Y | U | Y | Y | Y |
| Lewerenz et al., 2018 | Y | Y | Y | N/A* | Y | Y | Y | Y | Y |
| Markowitz et al., 2021 | Y | Y | Y | N | Y | Y | Y | Y | Y |

**Q1–Q9:** Q1, clear cause and effect; Q2, participants included in comparisons similar; Q3, participants received similar treatment/care other than the intervention; Q4, presence of a control group; Q5, multiple measurements before and after the intervention; Q6, complete follow-up or adequately described differences; Q7, outcomes measured in the same way; Q8, outcomes measured reliably; Q9, appropriate statistical analysis.

*Lewerenz et al. used within-participant comparison conditions rather than an independent parallel control group; therefore, the conventional independent-control criterion was considered not applicable.

**B.** JBI Critical Appraisal Checklist for Case Series

| Study | Clear inclusion criteria | Standard/reliable condition measurement | Valid identification methods | Consecutive inclusion | Complete inclusion | Demographics reported | Clinical information reported | Outcomes/follow-up reported | Appropriate statistics |
|---|---|---|---|---|---|---|---|---|---|
| Verezen et al., 1996 | Y | Y | Y | U | U | U | Y | Y | Y |
| Bertrand et al., 1997 | Y | Y | Y | U | U | U | Y | Y | Y |
| Vettard et al., 2004 | Y | Y | Y | U | U | U | Y | Y | Y |

**Abbreviations:** Y, yes; N, no; U, unclear; N/A, not applicable.

**Note:** The appropriate Joanna Briggs Institute (JBI) critical appraisal checklist was selected according to study design. Where reporting was insufficient to determine whether a criterion was met, the item was classified as unclear rather than inferred from information not reported.
